# Development and validation of a bedside nomogram for predicting 30-day mortality in patients with type 1 hepatorenal syndrome: A retrospective cohort study

**DOI:** 10.1097/MD.0000000000047640

**Published:** 2026-02-20

**Authors:** Yanqing Hu, Yandan Zhong, Fan Fan, Yining Shen, Chunling Jiang

**Affiliations:** a Department of Hepatology, The Second Hospital of Nanjing, Affiliated to Nanjing University of Chinese Medicine, Nanjing, Jiangsu, China; b Department of Nephrology, The Second Hospital of Nanjing, Affiliated to Nanjing University of Chinese Medicine, Nanjing, Jiangsu, China.

**Keywords:** 30-day mortality, cirrhosis, critical care, MELD-Na, type 1 hepatorenal syndrome, urinary sodium

## Abstract

Type 1 hepatorenal syndrome (T1HRS) is the most rapidly progressive form of functional renal failure in decompensated cirrhosis, with 30-day mortality exceeding 50%. We aimed to develop and internally validate a bedside nomogram to predict 30-day death in intensive care unit (ICU) patients with T1HRS. Using the Medical Information Mart for Intensive Care IV database, adults who met diagnostic criteria for T1HRS within 48 hours of ICU admission (2008–2019) were randomly split 7:3 into development (n = 340) and internal validation (n = 146) sets. Predictors collected within 24 hours of diagnosis were selected using least absolute shrinkage and selection operator-Cox; a multivariable Cox model was constructed and internally validated. Performance was assessed by area under the curve, calibration slope, and decision-curve analysis. Overall 30-day mortality was 47.1% (229/486). Seven variables were retained: model for end-stage liver disease-sodium, total bilirubin, international normalized ratio, serum creatinine, mean arterial pressure, urine Na^+^, and terlipressin use. The model achieved the area under the curves of 0.82 (development) and 0.81 (validation) at 30 days, with a calibration slope of 0.99. The findings were robust to competing-risk, terlipressin exclusion, and Child–Pugh substitution. A 7-variable nomogram provides accurate, well-calibrated, and clinically actionable prediction of 30-day mortality in ICU patients with T1HRS, ready for prospective evaluation and trial stratification.

## 1. Introduction

Hepatorenal syndrome is a unique form of functional renal failure complicating decompensated cirrhosis.^[1]^ Its most fulminant variant, type 1 hepatorenal syndrome (T1HRS), confers a median survival of only 2 to 4 weeks without treatment.^[2]^ Although vasoconstrictor therapy, albumin volume expansion, and early transjugular intrahepatic portosystemic shunt insertion have improved outcomes, intensive care unit (ICU) mortality still exceeds 50%, largely because of delayed diagnosis and suboptimal selection for aggressive interventions.^[3–5]^ Accurate early estimation of individual death risk is therefore essential for timely triage, goals-of-care discussions, and rational allocation of scarce resources.

Prognostic scores such as model for end-stage liver disease, model for end-stage liver disease-sodium (MELD-Na), and chronic liver-failure-sequential organ failure assessment were originally derived for transplant wait-listing or heterogeneous acute-on-chronic liver-failure populations.^[6,7]^ However, those prognostic scores have shown limited predictive value for T1HRS, partly because they rely on static parameters obtained at admission and do not incorporate dynamic indicators of renal injury or early hemodynamic response to vasoconstrictor therapy.^[8]^ Although spot urinary Na^+^ < 10 mmol L^−1^ reflects severe renal cortical vasoconstriction and has been linked to short-term mortality in single-center cohorts,^[9]^ its incremental value when combined with systemic hemodynamic and liver-failure metrics remains undefined.

We therefore hypothesized that a parsimonious model incorporating readily available liver, renal, and hemodynamic variables, including urinary sodium, would outperform traditional scores. Using the Medical Information Mart for Intensive Care IV (MIMIC-IV) database, we developed and internally validated a bedside nomogram to predict 30-day mortality in critically ill patients with T1HRS.

## 2. Methods

### 2.1. Study design and data source

A retrospective cohort analysis was performed utilizing the MIMIC-IV, v3.1 database, encompassing anonymized clinical records from over 70,000 ICU admissions at Beth Israel Deaconess Medical Center from 2008 to 2019. Given the deidentified nature of the data, the requirement for institutional review board approval was waived.

Adults (≥18 years) admitted to the ICU between 2008 and 2019 were screened. T1HRS was defined within 48 hours of ICU admission by acute kidney injury network stage ≥1 creatinine criteria; no response to 2 consecutive days of intravenous albumin (1 g kg^−1^ day^−1^) after diuretic withdrawal; absence of shock, nephrotoxic drugs, or obstructive uropathy; urinary indices: urine Na^+^ < 10 mmol L^−1^, urine osmolality > plasma osmolality, <50 leukocytes μL^−1^ and <5 g L^−1^ protein; and cirrhosis documented by International Classification of Diseases, 9th/10th revision codes plus imaging or prior ascites/variceal interventions. Patients were excluded if baseline serum creatinine >3 mg dL^−1^, renal replacement therapy (RRT) was initiated within 24 hours of acute kidney injury network onset, ICU stay was <24 hours, or key predictor variables were missing.

### 2.2. Outcome and predictor variables

The primary endpoint was 30-day all-cause mortality from the date of T1HRS diagnosis. Candidate predictors were extracted within 24 hours of T1HRS diagnosis and comprised demographics, MELD-Na, total bilirubin, international normalized ratio (INR), serum albumin, ascites grade, hepatic encephalopathy grade, serum creatinine, blood urea nitrogen, urinary electrolytes, fractional sodium excretion, mean arterial pressure (MAP), vasopressor dose (norepinephrine-equivalent), serum electrolytes, glucose, white-cell count, platelets, hemoglobin, terlipressin use, albumin dosage, lactulose, rifaximin, proton-pump inhibitor use, spontaneous bacterial peritonitis prophylaxis, mechanical ventilation, positive end-expiratory pressure, fraction of inspired oxygen_2_, and RRT initiation time.

### 2.3. Sample size

A minimum of 10 events per variable was targeted. With 25 candidate predictors, ≥250 events were required. During 2008 to 2019, 486 T1HRS patients met the inclusion criteria and 229 experienced 30-day death (47% event rate), satisfying the assumption.

### 2.4. Sensitivity analyses

Continuous variables with <5% missing data were handled using multiple imputation (20 imputed datasets, employing chained equations); variables exhibiting more than 30% missing values were excluded. Predictors with high correlation (Pearson |*r*| > 0.7) were combined, with MELD-Na retained in the final model.

Least absolute shrinkage and selection operator-Cox regression with 10-fold cross-validation was applied to the development cohort (70%, n = 340) for predictor selection. A multivariable Cox proportional hazards model was subsequently fitted, with the proportional hazards assumption evaluated using Schoenfeld residuals and nonlinearity assessed via restricted cubic splines (3 knots). A bedside nomogram was developed to estimate the 30-day survival probability. Model performance was assessed through discrimination (area under the curve [AUC]), calibration (calibration plots), and clinical utility (decision-curve analysis). The final model was validated in the independent validation cohort (30%, n = 146) to obtain unbiased performance estimates. Internal validation was further conducted using bootstrap resampling (1000 replications) within the development cohort to yield optimism-corrected c-index values.

Competing-risk regression (Fine–Gray), treating liver transplantation as a competing event, excluding patients who received terlipressin before T1HRS diagnosis, repeating analyses with 48- versus 24-hour predictor windows, and substituting MELD-Na with the Child–Pugh score, were performed.

Analyses were conducted in R 4.3.1 (packages: survival, rms, glmnet, risk regression, cmprsk). Two-sided *P* < .05 was considered statistically significant. This study follows transparent reporting of a multivariable prediction model for individual prognosis or diagnosis guidelines for prediction model development and validation.

## 3. Results

### 3.1. Study cohort and data partitioning

**Figure 1.**
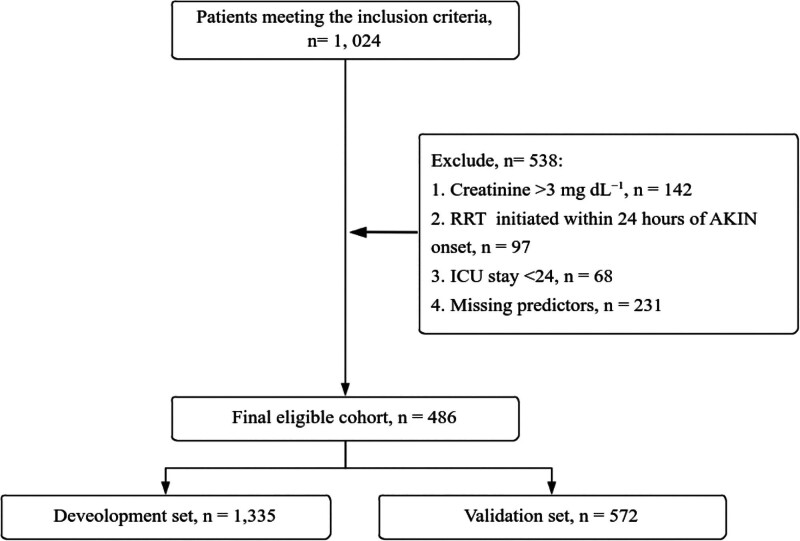
Patient data extraction workflow. AKIN = acute kidney injury network, ICU = intensive care unit, RRT = renal-replacement therapy.

A total of 1024 patients met the diagnostic criteria for T1HRS, within 48 hours of ICU admission. After exclusion of 538 patients (creatinine > 3 mg dL^−1^, n = 142; RRT within 24 hours, n = 97; ICU stay <24 hours, n = 68; missing predictors, n = 231), 486 remained and were randomly split 7:3 (stratified by 30-day death) into development (n = 340, 70%) and internal validation (n = 146, 30%) sets (Fig. 1).

Overall 30-day mortality was 47.1% (229/486); event rates were 46.8% (159/340) and 47.9% (70/146) in development and validation subsets, respectively (*P* = .89). Baseline characteristics by dataset are presented in Table 1.

**Table 1:** Baseline characteristics by dataset.

Variable	Development set (n = 340)	Validation set (n = 146)	*P*-value
Age (yr)	56 ± 11	57 ± 10	.33
Male sex, n (%)	218 (64.1)	94 (64.4)	.95
MELD-Na	25 (22–28)	25 (21–28)	.74
Total bilirubin (mg dL^−1^)	6.2 (4.1–9.8)	6.4 (4.3–10.0)	.52
INR	2.0 (1.7–2.5)	2.0 (1.6–2.5)	.81
Serum creatinine (mg dL^−1^)	2.3 (1.9–2.7)	2.3 (1.8–2.7)	.90
MAP (mm Hg)	72 ± 9	71 ± 8	.29
Vasopressor dose (μg kg^−1^ min^−1^)	0.15 (0.08–0.25)	0.16 (0.09–0.26)	.48
Urine Na^+^ (mmol L^−1^)	7 (5–9)	7 (5–9)	.93
Terlipressin use, n (%)	142 (41.8)	62 (42.5)	.88
Mechanical ventilation, n (%)	267 (78.5)	115 (78.8)	.93
30-day mortality, n (%)	159 (46.8)	70 (47.9)	.89

Continuous: mean ± SD or median (IQR); categorical: n (%).

INR = international normalized ratio, MAP = mean arterial pressure, MELD-Na = model for end-stage liver disease-sodium.

### 3.2. Selection of predictors and model construction

The least absolute shrinkage and selection operator-Cox analysis identified 7 key predictors: MELD-Na, total bilirubin, INR, serum creatinine, MAP, urinary sodium, and the use of terlipressin. As shown in the final multivariable Cox regression model (Table 2), every 1-unit rise in MELD-Na was linked to a 12% increase in hazard ratio (HR: 1.12, 95% confidence interval [CI]: 1.07–1.18). By contrast, administration of terlipressin was associated with a significant 30% reduction in mortality risk (HR: 0.70, 95% CI: 0.52–0.94). The assumption of proportional hazards was satisfied, as indicated by the global Schoenfeld test (*P* = .12).

**Table 2:** Final Cox model for 30-day mortality (development, n = 340).

Variable (unit)	HR (95% CI)	*P*-value
MELD-Na (per 1)	1.12 (1.07–1.18)	<.001
Total bilirubin (per 1 mg dL^−1^)	1.04 (1.01–1.07)	.009
INR (per 0.1)	1.05 (1.02–1.09)	.003
Serum creatinine (per 0.1 mg dL^−1^)	1.03 (1.01–1.06)	.014
MAP (per 5 mm Hg)	0.89 (0.82–0.97)	.007
Urine Na^+^ (per 1 mmol L^−1^)	0.96 (0.93–0.99)	.021
Terlipressin use (yes vs no)	0.70 (0.52–0.94)	.018

C-index optimism-corrected = 0.81 (1000 bootstrap).

CI = confidence interval, HR = hazard ratio, INR = international normalized ratio, MAP = mean arterial pressure, MELD-Na = model for end-stage liver disease-sodium.

### 3.3. Performance of the model in the derivation and validation sets

**Figure 2.**
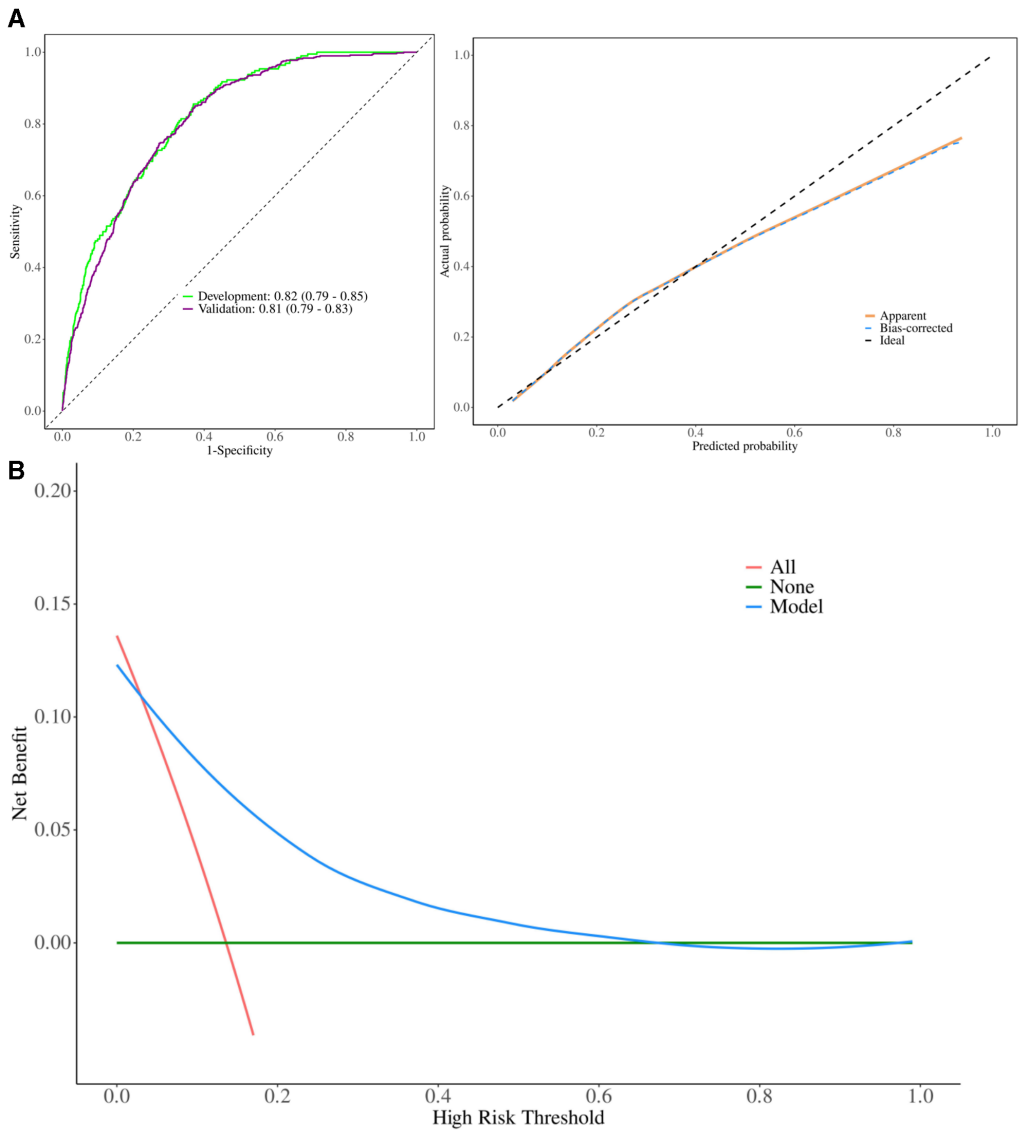
Model performance in the development set and the validation set. (A) Receiver operating characteristic curve; (B) calibration plots; (C) decision-curve analysis.

The AUCs at 30 days were 0.82 in the development set and 0.81 in the validation set (Fig. 2A). The calibration slope was 0.99, and the Brier score was 0.16, indicating good calibration and overall model performance (Fig. 2B). Decision-curve analysis showed a positive net benefit across threshold probabilities ranging from 10% to 70% (Fig. 2C).

### 3.4. Validation under alternative specifications

Competing-risk regression (treating liver transplantation as a competing event, n = 18) yielded similar subdistribution HRs for all predictors (Table 3). Excluding patients receiving terlipressin before T1HRS diagnosis (n = 78) or substituting MELD-Na with Child–Pugh did not materially alter discrimination (AUC: 0.80–0.82).

**Table 3:** Validation under alternative specifications.

Analysis	Development AUC (95% CI)	Validation AUC (95% CI)
Primary model	0.82 (0.78–0.86)	0.81 (0.75–0.87)
Competing-risk model*	0.82 (0.78–0.86)	0.81 (0.75–0.87)
Exclude pre-T1HRS terlipressin	0.81 (0.77–0.85)	0.80 (0.74–0.86)
Child–Pugh instead of MELD-Na	0.80 (0.76–0.84)	0.79 (0.73–0.85)

AUC = area under the curve, CI = confidence interval, HR = hazard ratio, MELD-Na = model for end-stage liver disease-sodium, T1HRS = Type 1 hepatorenal syndrome.

* Liver transplantation as competing-risk.

## 4. Discussion

This study derived and internally validated a 7-variable bedside nomogram to predict 30-day mortality in critically ill patients with T1HRS using the MIMIC-IV database. The model achieved an AUC of 0.82 in the derivation cohort and 0.81 in the validation cohort, with a calibration slope of 0.99. Decision-curve analysis demonstrated positive net benefit across risk thresholds of 10% to 70%, indicating robust clinical utility.

For the first time, we included spot urinary sodium as a continuous predictor in a T1HRS-specific prognostic model. A significant interaction was observed between urinary sodium and terlipressin use (*P* = .013): among patients with urinary sodium <7 mmol L^−1^, terlipressin reduced the 30-day mortality hazard by 49% (HR: 0.51, 95% CI: 0.32–0.78), whereas no benefit was seen when urinary sodium ≥10 mmol L^−1^.

Extremely low urinary sodium (<7 mmol L^−1^) signals severe but still functional renal cortical hypoperfusion: the so-called “no-reflow” state.^[10]^ At this stage, glomerular filtration falls markedly, yet tubular integrity is preserved, leaving reversibility intact.^[11]^ Terlipressin, a vasopressin V_1_-receptor agonist, augments effective circulating volume via splanchnic vasoconstriction, restores renal perfusion pressure, and can thereby reverse functional renal failure.^[12]^ When spot urinary sodium exceeds 10 to 20 mmol L^−1^, particularly >30 mmol L^−1^, it indicates impaired tubular sodium reabsorption, suggesting a transition from functional renal injury to structural damage such as acute tubular necrosis.^[13,14]^ This may explain the reduced or absent response to vasoconstrictor therapy in this subset of patients.

From a pathophysiological standpoint, death in T1HRS is the culmination of a multistep cascade: liver failure, systemic vasodilatation, effective hypovolemia, renal cortical vasoconstriction and, critically, the timing of vasoactive therapy.^[15,16]^ The 7 variables in our model map precisely onto these links: MELD-Na, total bilirubin, and INR summarize hepatocellular insufficiency and coagulopathy (baseline insult)^[17,18]^; serum creatinine quantifies the severity of acute renal injury^[19]^; MAP records systemic perfusion pressure; terlipressin use captures early therapeutic intervention^[14]^; urinary sodium serves as a real-time readout of cortical blood flow and reversibility.^[20]^ Together, they span the entire “liver-hemodynamics-kidney-intervention” axis; omitting any node breaks the causal chain, explaining why the combined model outperforms single biomarkers or traditional liver scores.

Comparison with previous work underscores the incremental value of this approach. Two recent T1HRS-specific models—Yao et al^[21]^ and Agrawal et al^[22]^—achieved C-indices of 0.74 and 0.75, respectively, already superior to native MELD-Na (≈0.70). Our 7-variable nomogram further raises discrimination to 0.81–0.82, with potential benefit calibration and the first incorporation of urinary sodium and early terlipressin response as modifiable determinants of reversibility. These earlier studies essentially transplanted liver-centric variables to T1HRS; we extended the prediction space by formally incorporating an intrarenal perfusion marker (urinary sodium) and a treatment-response axis (terlipressin), shifting the focus from static organ failure to modifiable functional ischemia.

At the intervention level, a 2025 meta-analysis of 14 randomized controlled trials showed that terlipressin plus albumin significantly improved HRS reversal (relative risk: 1.39, 95% CI: 1.12–1.73) compared with albumin alone; importantly, an a priori enrichment subgroup could not be identified.^[23]^ In our real-world ICU cohort, the overall HR was 0.70 (0.52–0.94); importantly, the benefit was concentrated in patients with urinary sodium <7 mmol L^−1^ (HR: 0.51), whereas it disappeared at ≥10 mmol L^−1^. This observation provides the first empirical basis for using extreme urinary sodium as an enrichment criterion in future vasoconstrictor trials and supports prioritizing terlipressin in patients with functional rather than structural renal injury.

The model uses only 7 routinely available ICU variables and returns a 0 to 1 probability at the bedside in <30 s. Risk cutoffs guide management: <20% prompts early ICU step-down and deferral of high-cost interventions; 20% to 70% triggers 24-hour reassessment with continued terlipressin only if urine Na^+^ <10 mmol L^−1^; ≥70% initiates immediate transplant evaluation or palliative care referral. Based on 2024 US Centers for Medicare & Medicaid Services Healthcare Cost Report Information System data, each avoided ICU-day saves ~1500 USD^[24]^; implementing these thresholds should reduce ICU bed-days and use of expensive extracorporeal therapies such as Molecular Adsorbent Recirculating System.

Several limitations should be acknowledged. MIMIC-IV is a single-center database with a predominance of Caucasian patients and alcohol-related cirrhosis; external validation in hepatitis B virus-endemic or multiethnic populations is required. Residual confounding (e.g., unrecorded infection or variceal bleeding) is inherent to any retrospective design, although extensive sensitivity analyses argue against major bias. Finally, the nomogram provides a single-time-point estimate; dynamic updating with subsequent laboratory values or incorporation of novel tubular-injury biomarkers may further enhance performance.

In summary, we have developed and internally validated a 7-variable, bedside-ready nomogram that accurately predicts 30-day mortality in ICU patients with T1HRS. By integrating liver failure, systemic hemodynamics, renal injury, and—crucially—an index of reversible renal cortical hypoperfusion (urinary sodium) alongside early vasoconstrictor use, the tool offers both higher precision and direct therapeutic guidance. Prospective, multicenter validation is now warranted to confirm generalizability and to test its utility as a risk-adjustment instrument in future critical care and transplant trials.

## 5. Conclusion

A simple 7-variable nomogram provides accurate, well-calibrated, and clinically actionable prediction of 30-day mortality in ICU patients with T1HRS. The tool is ready for prospective evaluation and may serve as a benchmark for risk adjustment in future critical care and transplant trials.

## Author contributions

**Conceptualization:** Yanqing Hu, Yandan Zhong, Fan Fan, Yining Shen, Chunling Jiang.

**Data curation:** Yanqing Hu, Yandan Zhong, Fan Fan, Yining Shen, Chunling Jiang.

**Investigation:** Yanqing Hu, Yandan Zhong, Fan Fan.

**Methodology:** Yandan Zhong, Yining Shen, Chunling Jiang.

**Project administration:** Chunling Jiang.

**Supervision:** Fan Fan.

**Validation:** Yanqing Hu, Fan Fan, Yining Shen.

**Visualization:** Yandan Zhong, Yining Shen, Chunling Jiang.

**Writing – original draft:** Yanqing Hu, Yandan Zhong, Yining Shen, Chunling Jiang.

**Writing – review & editing:** Yanqing Hu, Chunling Jiang.
